# Advancements in Three-Dimensional Super-Resolution Ultrasound Imaging

**DOI:** 10.1002/jum.16682

**Published:** 2025-03-12

**Authors:** Debabrata Ghosh, Kenneth Hoyt

**Affiliations:** Department of Electronics and Communication Engineering, Thapar Institute of Engineering and Technology, Patiala, India; Department of Biomedical Engineering, Department of Small Animal Clinical Sciences, Texas A&M University, College Station, Texas, USA; Department of Small Animal Clinical Sciences, Texas A&M University, College Station, Texas, USA

**Keywords:** contrast-enhanced ultrasound imaging, microbubble contrast agents, microvascular networks, singular value decomposition, super-resolution ultrasound imaging, ultrasound, volumetric imaging

## Abstract

The lack of sensibility of traditional ultrasound (US) imaging to the slow blood flow in small vessels resulted in the development of microbubble (MB) contrast agents. These MBs are given intravenously, and US imaging can detect them quite effectively. This noninvasive imaging method, known as contrast-enhanced US (CEUS), now makes it possible to accurately assess tissue perfusion and blood flow. Though CEUS offers several benefits, diffraction restricts the spatial resolution of all US imaging systems to length scales equal to roughly half the wavelength of the transmitted US beam. Based on individual MB detection and localization, the recently developed super-resolution US (SRUS) imaging method has shown unprecedentedly high spatial resolution exceeding the physical diffraction limit. It is now possible to visualize the microvasculature beyond the diffraction-limited resolution by localizing spatially isolated MBs across several frames. The highest resolution possible at clinical US frequencies can be on the order of several micrometers when tissue and probe motion are not present. Enhancing the functional study of tissue microvascular networks with structural data could lead to improved disease management. Through the localization and tracking of MBs, SRUS may reconstruct images of the microvasculature with resolution exceeding the diffraction limit in both 2-dimensional (2D) and 3-dimensional (3D) space. In contrast to the 2D approach, 3D SRUS imaging does not suffer from out-of-plane motion and can offer volumetric coverage with super-resolution in all three dimensions. Research has used two primary methods for 3D SRUS imaging including arrays that can electronically gather volumetric information or mechanically scanning the volume with a linear probe to produce a stack of 2D SRUS images. This manuscript aims to offer a comprehensive review of 3D SRUS imaging, clarifying methodologies, clinical applications, and notable challenges that could motivate future research and help facilitate clinical translation.

Microvascular networks play a pivotal role in several critical diseases, including tumor progression, chronic kidney disease, atherosclerotic plaque development, and various cardiovascular and neurological disorders. Microvascular changes serve as a vital diagnostic index for these conditions, emphasizing the need for advanced imaging modalities capable of detailed microvascular observation.^1^

Medical imaging modalities like magnetic resonance imaging (MRI), computed tomography (CT), and ultrasound (US) are ubiquitous in the modern clinical setting. US has several notable attributes, including real-time imaging capability, portability, patient safety, and cost-effectiveness.^2^ Traditional US methods rely on transducer manipulation to capture signals and produce 2-dimensional (2D) images. US waves can travel deep distances on the order of centimeters without significant loss of integrity; yet, their spatial resolution is inherently limited to approximately half of the wavelength (0.1–1 mm), restricting the visibility of finer details above 100 μm at clinical US frequencies (e.g., 8 MHz).^3^

The advent of 2D super-resolution US (SRUS) has overcome some of these limitations by employing techniques such as microbubble (MB) contrast agent localization. SRUS is able to generate spatially resolved maps by imaging isolated MB signals from the backscattered US data.^4^ This method allows for visualization of microvascular detail beyond the physical diffraction limit^5^ enabling detection of vessels of a scale of tens of micrometers in diameter.^6^

The 2D SRUS faces inherent limitations in that super resolution cannot be achieved in the elevational direction, and out-of-plane motion can only be compensated for movements smaller than the elevational beamwidth of the transducer. These constraints have spurred the development of 3-dimensional (3D) SRUS imaging techniques, which can overcome such limitations and enhance the visualization of microvascular structures.^7^ To achieve 3D visualization, innovative approaches such as orthogonal probe arrangements or mechanically translated linear arrays have been employed.^8^ To circumvent the complexities of mechanical scanning, matrix array probes have been increasingly used.^9^

Transitioning from 2D to 3D in SRUS imaging presents a set of challenges related to system complexity and the sophisticated tracking of individual MB positions.^10^ While 2D SRUS generally offers better image quality, 3D SRUS provides volumetric coverage, necessitating advanced reconstruction and visualization techniques.^11^ Modern 3D SRUS uses 2D matrix arrays to expedite the US imaging process, though cost considerations remain a factor, with matrix probes requiring more elements than their 1D counterparts.^10,12^ Figure 1 illustrates the technological progression from traditional US methods to the 3D SRUS, showcasing the significant advancements made in imaging capabilities.

The motivation for this review arises from the rapid progress in 3D SRUS imaging and its transformational potential in medical diagnostics. It is crucial to systematically review and integrate the most recent advancements, challenges, and future avenues as this technology evolves. This review aims to offer a comprehensive assessment that not only informs the medical community about the current abilities and applications of 3D SRUS, but also identifies the areas of knowledge and technology gaps that may inspire future research. This work aims to emphasize the significance of ongoing investment and innovation in the field by examining recent advancements and their clinical impacts. It seeks to promote a deeper understanding that could expedite the translation of 3D SRUS from research environments to widespread clinical use.

The rest of this article is structured as follows: The next section presents the study selection process; Methodologies for 3D SRUS Imaging section provides a detailed review of current 3D SRUS imaging techniques and their performance; Clinical Applications section outlines the clinical applications of 3D SRUS; and Outstanding Challenges section discusses the outstanding challenges in the field; and the last section concludes the article.

## Study Selection

The systematic review began with a comprehensive search across multiple popular electronic databases, including PubMed, Google Scholar, Web of Science, IEEE Xplore, and the SPIE Digital Library. The search strategy employed a combination of keywords to encompass the entirety of the field: “3-dimension and super-resolution ultrasound,” “3-dimension and SRUS,” “3-dimension and ultrasound localization microscopy,” “3-dimension and ULM,” “3D and super-resolution ultrasound,” “3D and SRUS,” “3D and ultrasound localization microscopy,” “3D and ULM,” “volumetric and super-resolution ultrasound,” “volumetric and SRUS,” “volumetric and ultrasound localization microscopy,” and “volumetric and ULM.” This initial query returned a total of 128 scientific articles.

Following the search, 51 articles were excluded due to the removal of duplicates. An additional round of screening was conducted to exclude 49 articles based on additional criteria, including relevance to the topic, appropriate study design, availability of full text, and publication in English. Ultimately, 28 studies were considered appropriate and were included in this systematic review. These selected studies are crucial to understanding the latest developments in 3D SRUS imaging and lay the foundation for future research that must consider several additional dimensions for further exploration in this rapidly evolving field.

## Methodologies for 3D SRUS Imaging

**Figure 2.**
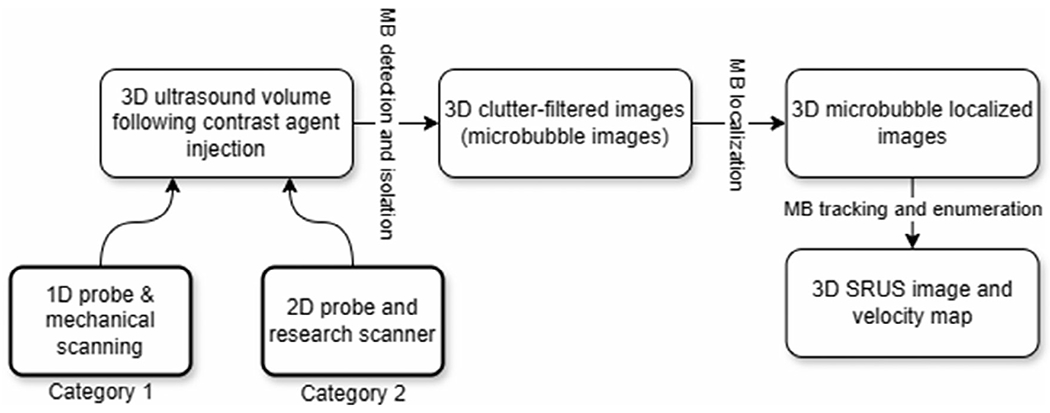
Schematic of the 3D SRUS imaging technique.

Significant developments have been made in the field of 3D SRUS imaging, with the current state-of-the-art techniques being categorized into two main groups. The first category is based on traditional 1-dimensional (1D) array probes that acquire 2D data at different imaging planes while translating it mechanically. Subsequently, the computation of 2D SRUS is performed in each imaging plane, followed by the utilization of 3D reconstruction procedures to get the intended 3D SRUS imagery. On the other hand, the second group covers methodologies that utilize US transducers and scanners that are specifically designed to acquire volumetric data. After the acquisition of this data, computational methods are employed to produce 3D SRUS images. Figure 2 illustrates the schematic representation of the two main categories of 3D SRUS. Beyond the first category, an alternative strategy involves 3D MB localization and subsequently 3D SRUS imaging using a pair of 1D US transducers positioned at 90°,^13^ connected to an “active” system and a “passive” system that were temporally synchronized.

### 3D SRUS by Mechanically Scanning a 1D Transducer

The combination of a 1D transducer and a mechanical system capable of shifting the transducer has facilitated the initial advancement of 3D SRUS imaging. A 1D linear array US transducer has the capability to generate a 3D US image. The process involves the conversion of a series of 2D SRUS frames into a comprehensive 3D SRUS volume using 3D reconstruction. One approach involves the utilization of a mechanical scanning system. A 3D mechanical probe,^12^ housed in a compact casing, uses an internal stepper motor to maneuver an ultrasonic linear array around the region of interest (ROI). This motor facilitates tilting, rotating, and linear movements, enabling the acquisition of multiple 2D US images around the ROI.^14^ However, these probes have not been employed for 3D SRUS studies. Another variant of the mechanical scanning system uses an external motorized setup, like a precision stage, to position the US probe. This system guides the transducer along a predetermined path and orientation around the ROI, ensuring the consistent spacing between the acquired 2D US frames and maintaining their precise position and orientation in relation to a reference frame. The later technique has been extensively reported in the literature.^15,16^

Errico et al^15^ used US localization microscopy (ULM) to produce SRUS images of microvascular structures and blood flow velocity maps in the rat brain. Leveraging ultrafast frame rates of 500 frames per second, they achieved rat transcranial whole-brain (5 × 3 mm, width × depth) imaging within 10 minutes of data acquisition. The 3D SRUS imaging was facilitated using a programmable clinical scanner and a custom-built probe (160 elements, 20.3 MHz central frequency) paired with a motor, systematically scanning each coronal plane. Following a singular value decomposition (SVD)-based clutter filtering and deconvolution-based localization, individual MBs were tracked in axial position and in depth, allowing the reconstruction of detailed vascular structures with a spatial resolution reaching just a few micrometers at several millimeters depth.

Lin et al^17^ explored the potential of 3D ULM for discerning microvascular morphology features associated with tumor angiogenesis. They used a customized programmable US system equipped with a linear probe (128 elements, 7.8 MHz central frequency) and synchronized it with a mechanical scanning system. ULM processing involved high-pass spatiotemporal SVD filtering, MB decorrelation detection, hysteresis thresholding, and centroid detection. Following this, 3D SRUS images were derived by compiling the maximum intensity projection from all ULM image slices. Using a frame rate of 500 Hz, their system achieved a complete 3D scan of a rat fibrosarcoma tumor (5 mm) within 11 minutes of data acquisition and helped visualize complex microvascular patterns at a resolution of tens of microns.

**Figure 3.**
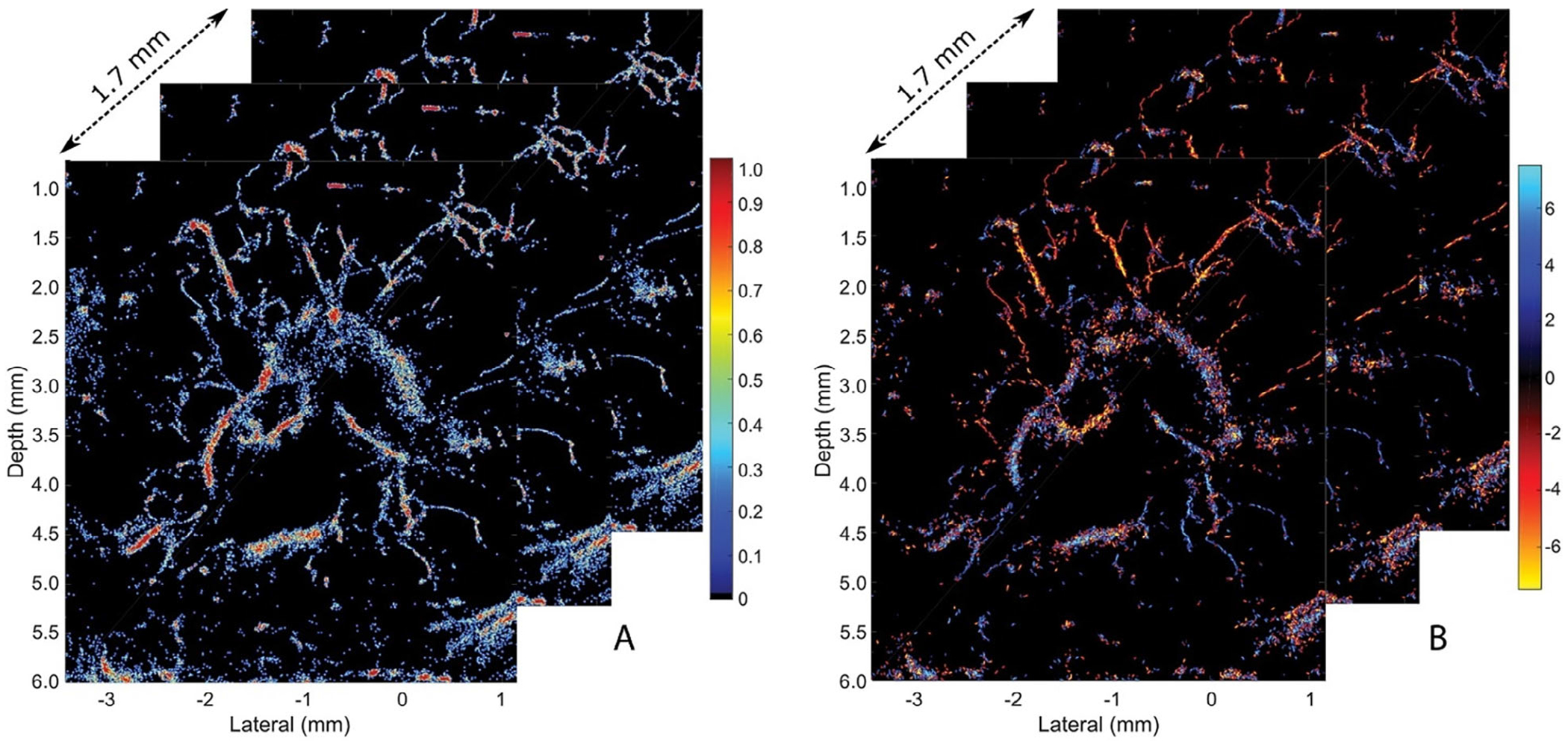
Images show a 2D cross-section of the popliteal lymph node. **A**, SRUS image where the color bar corresponds to the normalized number of localized microbubbles (MBs). **B**, SRUS velocity map where blue indicates blood flow toward the transducer and red indicates flow going away.^18^

Zhu et al^18^ studied the capabilities of 3D SRUS for noninvasively visualizing the microvascular structure and blood flow within rabbit lymph nodes (Figure 3). The 3D US data were captured using an US system equipped with a linear imaging probe (128 elements, 18.5 MHz central frequency) that mechanically scanned the target area. A complete 3D scan of 1.7 mm distance was achieved within 40.8 seconds of data acquisition. Individual MBs were localized and tracked to construct 3D SRUS images and super-resolved velocity maps by projecting the maximum intensity from all 2D SRUS image slices. Results showed the potential of this method to visualize the microvasculature in lymph nodes with spatial resolutions less than 80 μm.

Oezdemir et al^6,19^ employed a high-frequency preclinical US scanner equipped with a linear array transducer (center frequency 21 MHz) to collect contrast-enhanced US (CEUS) images in a developing chicken embryo model. The transducer was mechanically scanned through a tissue volume of interest, capturing CEUS images at each incremental position. Using a frame rate of 152 Hz, it took 3.5 minutes of data acquisition for imaging each volume. SVD-based speckle filtering, connected component identification, and centroid detection were used to generate SRUS for each cross-sectional plane. These SRUS images were then resampled to reconstruct an isotropic 3D SRUS volume. Results showed increased spatial resolution (about 20 μm) and improved quantification of microvascular features from the 3D SRUS images.

The same group^16^ investigated the potential of 3D SRUS in assessing the early changes in cancer after treatment with a vascular disrupting agent (VDA). The imaging data was captured from a breast cancer-bearing mice using a preclinical US system equipped with a linear array transducer (center frequency 15 MHz). Stacks of US images were collected by mechanically moving the transducer across the tumor. A complete 3D scan of a 6-mm thick volume was achieved within 3.5 minutes of data acquisition at a frame rate of 463 Hz. Each stack was processed to create SRUS images. Following, 3D SRUS volumes were reconstructed. The 3D SRUS-derived results showed significant differences in mean microvascular density measurements before and after VDA treatment.

### 3D SRUS Using Specialized Scanners and Probes

The precision of MB localization fails when MBs are positioned too close to each other (within a few wavelengths) causing their point spread functions (PSFs) to interfere with each other. Consequently, only a limited number of MBs can be distinguished in an individual image, resulting in the need for lengthy acquisition times to unveil micrometer-sized vascular structures within each plane.^20^ Thus, a plane-by-plane SRUS imaging of an entire volume, as employed by the techniques reviewed so far, remains a challenging task. Furthermore, the out-of-plane movement of MB during 2D imaging with a 1D transducer can introduce biases in MB location and velocity estimation.^21^ To address these challenges, recent advancements have shifted towards employing specialized scanners and probes. The use of 2D matrix probes, equipped with a dense array of elements, allows for the capture of volumetric US data, facilitating MB tracking in 3D and potentially compensating for the out-of-plane motion issues. To achieve high-quality 3D imaging while reducing system complexity, various strategies have been explored within this category, including synthetic aperture, sparse arrays, and row-column-addressed matrix arrays. These techniques aim to provide a comprehensive and accurate representation of ROIs, overcoming the limitations of traditional 2D imaging and offering a volumetric view of microvascular structures. Desailly et al^3^ employed a 128-channel clinical US scanner connected to a 1.75 MHz 2D matrix transducer to perform 3D ultrafast ULM in an in vitro study. At a frame rate of 1 kHz, a complete 3D scan of the volume of interest required 2.5-minute data acquisition. Following a differential imaging to isolate the MB signals, two parallel series of 64 transducer elements were used to localize MBs along the elevation axis. While using parallel matrix elements along with plane wave imaging, MBs appeared paraboloid. These paraboloids were fitted using an ultrasonic time-of-flight model to determine the exact source of the MB. Results showed a resolution improvement of 20 times finer than the US imaging wavelength.

In a trans-skull imaging setup, O’Reilly and Hynynen^22^ mapped individual MBs flowing through a tube phantom using a sparse hemispherical receiver array (128 elements) and a passive beamforming algorithm. US was transmitted using a subset of 128 elements from a larger 1372-element hemispherical transcranial therapy array, with a center frequency of 306 kHz and driven by a 128-channel clinical US system operating at a 2 kHz pulse repetition frequency (PRF). A 10 × 10 × 10 cm^3^ volume took 15 minutes to capture. Reference scans were subtracted from MB measurements to suppress clutter. For creating 3D SRUS images, a 3D Gaussian shape was fitted to each frame containing a single discernible source, followed by merging the normalized frames and selecting the maximum pixel projection. In their later study,^23^ Foroozan et al compared the aforementioned curve fitting-based SRUS imaging method with a deconvolution-based method for post-processing MB images generated through passive acoustic mapping. Unlike the former, this approach iteratively processed multi-frame images to estimate pixel intensities in the SRUS image without the necessity of localizing each MB. It assumes prior knowledge of the PSF, approximated as a Gaussian curve resembling the beam response of the hemispherical transducer array employed in their previous study.

In an in vitro study, Harput et al^7^ achieved 3D SRUS imaging using a density-tapered sparse array to reduce the number of channels and data size. They selected 512 elements (center frequency: 3.7 MHz) from a 2D matrix array to create two sparse arrays (256 elements each) connected to two 256-channel systems. Using compounded plane wave US imaging with a PRF of 2500 Hz, they achieved 3D SRUS of two touching subwavelength tubes within a 120-second image acquisition time. The same group developed a two-stage motion correction technique^24^ for 3D motion correction during 3D SRUS imaging. High volumetric rate US acquisitions were used for motion estimation, employing a wire phantom. The capability of this method was demonstrated through a 3D microvascular flow simulation, effectively compensating for handheld probe motion. Following SVD-based filtering, motion estimation was conducted on the 3D B-mode US data, with MB locations being corrected accordingly. The results indicated that the two-stage motion correction method significantly reduced the average localization error from 18 to 136 μm. In their subsequent study,^8^ utilizing a PRF of 4.5 kHz, they accomplished the 3D SRUS within 12 seconds of acquisition. Following SVD-based clutter filtering and MB localization with a precision of 18 μm, nearest neighbor-based tracking was performed to map super-resolved flow velocity. The obtained 3D SRUS images and velocity maps were further processed^5^ for quantitative microvessel analysis. However, despite these advancements, the use of sparse arrays necessitates many transducer channels to mitigate grating lobes, which often lead to degraded PSF and poor SNR.^9^

Christensen et al^4^ demonstrated 3D SRUS imaging of a twisted microvessel phantom using a clinical US system connected with a 3040-element 2D matrix array transducer. The system operated at a 1.25 MHz transmit frequency, capturing data at a frame rate of 54 Hz, and requiring 11 seconds to capture each 594 volumes. After applying a rolling background subtraction-based clutter filtering, MB localization in 3D was performed by identifying the intensity-weighted centers of mass. The 3D SRUS was obtained by rendering estimated MB locations as ellipsoids in a 3D space. Intensity cross-correlation was adopted between successive frames to track MBs in 3D.

Jensen et al^9,25^ developed a volumetric SRUS imaging method using a 3 MHz piezoelectric transducer row-column array connected to an experimental scanner. The method used a synthetic aperture scan sequence with 64 emissions (32 positive, 32 negative) for pulse inversion imaging, achieving a volume rate of 156 Hz at a 10 kHz PRF. Only 62 elements were used during reception, allowing for implementation on standard US consoles and generating limited data. The SRUS processing involved beamforming the received backscattered US signals, performing background subtraction, and then estimating MB/scatterer locations by identifying local maxima. Precision was tested using point and microscale flow phantoms, yielding localization precisions of (20.7, 19.8, 9.1) μm (*x, y, z*) and radial precisions of 16.5 μm (*y–z*) and 23 μm (*x–z*). In a follow-up study, the Ommen et al^26^ created a 3D printed microscale phantom with precisely positioned US scatterers and a translation stage to calibrate a SRUS imaging pipeline. They compared scatterer distances measured from their SRUS imaging pipeline to the phantom’s design distances. The variability in scatterer positions across 640 volumes were used as an estimate of the imaging pipeline’s precision. Notably, row-column arrays, though reducing channel counts, suffer from strong side lobes that distort the PSF in the nearfield, increasing the possibility of misidentifying side lobes as distinct MBs.

Heiles et al^20^ exploited volumetric ultrafast US imaging for 3D SRUS using a 32 × 32 9-MHz matrix-array probe, connected to four synchronized 256-channel US systems, to image a complex 3D structure. The systems were synchronized to receive with 512 multiplexed channels. Beamformed MB signals were initially processed through an SVD-based clutter filter and then deconvolved to localize MBs in 3D space. The Munkres assignment method was used for particle pairing and tracking, and a Lagrangian approach was employed for velocimetry. This method accurately represented 3D structures as small as 230 μm and provided a sufficient volume rate (500 Hz) to describe MB velocities between 2.5 and 150 mm/second, reducing the maximum acquisition time to 12 seconds.

Lok et al^11^ addressed the 3D SRUS’s complexity by using a subaperture process, reducing received channel counts while slightly lowering the frame rate. A 256-channel system and a 1024-channel matrix probe were used to transmit plane wave US signals at specific angles, and data was received through four 256-channel apertures. In a flow phantom experiment, after SVD-based clutter filtering, MBs were localized using normalized cross-correlation with a known 3D PSF. The 3D bipartite graph-based tracking was used for MB tracking. Their future work^21^ involved 3D motion registration, separation of MB data into datasets with sparser MBs, and Kalman-filter-based tracking. The technique revealed microvasculature structures as small as 52 μm during chicken embryo brain imaging.

To address the challenge of a large main lobe and substantial side lobes in 3D SRUS studies with standard delay and sum (DAS) beamforming, Yan et al^10^ introduced adaptive weight-based coherence beamformers, including a 3D coherence beamformer based on channel signal variance. A 256-channel system connected with a 32 × 32 matrix probe (7.8 MHz central frequency) was used to transmit plane wave US signals, with data received through four 256-channel apertures. After SVD-based clutter filtering and beamforming, 3D normalized cross-correlation (3DNCC) with a known PSF was performed. Peak identification was then employed for MB localization. Using simulation, in vitro, and *in vivo* (rabbit kidney) experiments, it was demonstrated that these beamformers can significantly improve the localization of densely distributed MBs.

In addressing the need of numerous acquisition channels in volumetric ULM, Chavignon et al^27^ implemented three different subaperture multiplexing methods using a 32 × 32 probe (7.8 MHz center frequency) and a single 256-channel research US scanner. Volumes were acquired with 5 compounded plane waves. These methods were evaluated *in silico*, in vitro, and for rat brain micro-angiography. SVD-based clutter filtering, local maxima detection, and a 3D radial symmetry-based localization were employed for localizing MBs, which were subsequently paired using a Kuhn-Munkres based algorithm, and their trajectories were projected onto a rendering volume. In the rat brain, 100,000 volumes were acquired in 7 minutes, revealing vessels as small as 31 μm with flows ranging from 4.3 to 28.4 mm/second. In a subsequent study,^28^ Chavignon et al explored the capability of their 3DULM imaging technique to distinguish between ischemic and hemorrhagic stroke microvascular patterns in rats. It was shown that the microvascular diffusion index (MDI) derived from 3DULM could effectively differentiate between these stroke subtypes during their early phases.

**Figure 4.**
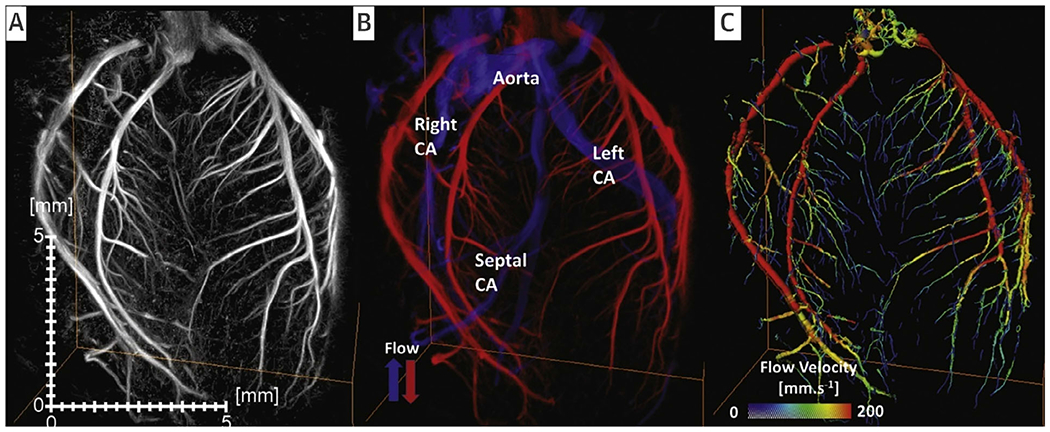
Example of 3-dimensional (3D)-coronary ULM (CorULM) of a perfused beating rat heart. **A**, 3D-MB density map; **B** directional blood flow velocity map that identifies the arterial (red) and venous (blue) flow and **C**, 3D-flow velocity distribution. CA, coronary artery.^30^

3D ultrafast ULM was conducted using a 32 × 32 probe (center frequency 9 MHz) connected to four synchronized research scanners (256-channel each), enabling whole brain microvasculature mapping in mice.^29^ For transmission, a coherent compounding of 16 plane wave images was employed, achieving a volume rate of 750 Hz with a total data acquisition time of 15 minutes for the whole brain. Following SVD-based clutter filtering, MBs were localized through local maxima detection and polynomial fitting, with their trajectories projected onto a rendering volume. An iterative intensity-based registration algorithm was used to align the vascular maps with the Allen atlas. This technique allowed imaging at a spatial resolution of 20 μm and tracking flow velocities ranging from 2 to 100 mm/second. In their follow-up study,^30^ Demeulenaere et al used the same setup for 3D coronary ULM on perfused rat hearts and anaesthetized rats (Figure 4), achieving a volume rate of 2220 volumes/second with 9 plane waves at a 20 kHz PRF. Multiscale 3D motion correction was applied to the same ULM processing, and corrected MB coordinates were aggregated into a detailed 3D vascular volume. Microvascular structures finer than 20 μm and flow velocities ranging between 10 and 300 mm/second were resolved.

To eliminate the need for pauses during data acquisition in 3D ULM, McCall et al^31^ utilized the Vantage buffer system in a unique way that separates data acquisition and data transfer. This asynchronous acquisition and transfer of data allowed full volumetric acquisition in rat brains within 200 seconds (500 volumes per second). A 1024-channel system, comprising four programmable US systems, connected with a 32 × 32 matrix array probe with a 7.81 MHz center frequency was used to acquire data with a 5-angle plane-wave compounding scheme. Following an SVD-based clutter filtering, MBs were localized and tracked using the Hungarian algorithm, achieving a resolution of 31 μm in 3D.

A 3D ULM imaging method was developed by Lei et al^32^ to visualize the ocular microvasculature in rabbits, utilizing a 32 × 32 matrix array transducer with an 8 MHz center frequency, synchronized across four programmable US research platforms. Through the implementation of block-wise SVD spatiotemporal clutter filtering and block-matching 3D denoising, flowing MB signals were extracted, localized, and tracked in 3D space, revealing vessels as small as 54 μm and identifying morphological abnormalities such as retinal detachment. Three hundred volumes were acquired at a rate of 500 volumes per second, employing delay-and-sum beamforming and 3D nonrigid image registration to estimate tissue motion and correct MB positions.

Table 1 presents a comprehensive summary of important studies on 3D SRUS, highlighting key aspects of each work for comparison. This comparative review intends to offer a thorough understanding of the current state of 3D SRUS research, emphasizing both the diversity of methodologies and the technological intricacies within the discipline.

## Clinical Applications

The advent of 3D SRUS has revolutionized the field of medical imaging by providing unprecedented insights into the microvascular architecture and flow dynamics of various tissues and organs. Following are the significant clinical applications of 3D SRUS, as demonstrated in the reviewed literature, highlighting its transformative potential across different medical specialties.

### Neurovascular Imaging (Transcranial Brain Imaging^15,22,27,29–31^)

Ultrafast frame rates have enabled noninvasive subwavelength structural imaging and hemodynamic quantification of cerebral microvessels, facilitating whole-brain imaging within short acquisition times. This advancement is crucial for understanding and diagnosing neurological diseases such as stroke, arteriosclerosis, and potentially degenerative diseases by mapping the microcirculation in depth in living tissue, including through the intact skull for longitudinal studies.

### Oncology (Tumor Angiogenesis^17^)

The ability to image complex microvascular patterns associated with tumor angiogenesis at a resolution of tens of microns represents a significant leap in cancer diagnostics. This technology allows for the detection of biomarkers of cancer based on the microvascular “fingerprint” of malignant angiogenesis, offering a novel approach to identifying and monitoring tumor progression and response to therapies.

### Developmental Biology (Embryonic Development [^6,19^] and Brain Imaging^21^)

The application of 3D SRUS in imaging developing chicken embryos and chicken embryo brains has improved the quantification of microvascular networks, providing a validation framework for analyzing microvascular morphology. This approach has shown better accuracy in vessel diameter quantification compared to traditional B-mode US, enhancing our understanding of embryonic development and vascular formation.

### Oncology Treatment Monitoring (Breast Cancer Treatment^16^)

Monitoring early changes in breast cancer after treatment with vascular-disrupting agents (VDAs) using 3D SRUS has shown potential in detecting early tumor changes following treatment. This method could significantly impact the assessment of therapeutic efficacy and the adjustment of treatment strategies based on microvascular responses.

### Lymphatic System Imaging (Lymph Node Microcirculation^18^)

Variations in lymph node microcirculation, which can indicate metastasis, have been successfully visualized using 3D SRUS. This noninvasive technique allows for the visualization and quantification of lymph node microvascular structures and blood flow dynamics, offering a new avenue for the clinical detection of lymph node metastasis.

### Cardiovascular Imaging (Coronary Microcirculation^30^)

Direct assessment of the coronary microcirculation has been achieved beyond the acoustic diffraction limit, providing detailed visualization of the coronary vasculature in beating hearts. This breakthrough has implications for understanding coronary artery disease and evaluating the effects of therapeutic interventions on microvascular flow.

### Vascular Imaging and Disease Diagnosis (Stroke Discrimination^28^)

Early discrimination between ischemic and hemorrhagic stroke using 3D transcranial ULM could revolutionize stroke diagnosis and treatment, offering rapid and accurate diagnoses that complement existing imaging modalities like CT and MRI.

### Ophthalmology (Ocular Microvasculature^32^)

The development of 3D ULM for visualizing the ocular microvasculature in rabbits showcases the potential for diagnosing ocular diseases by revealing morphological and hemodynamic changes in the eye microvasculature.

### Renal Imaging (Renal Microcirculation^10^)

Utilizing advanced 3D SRUS imaging techniques has significantly enhanced the visualization of renal microvascular structures in rabbits. The resultant SRUS images showcased a marked improvement in spatial resolution and image quality, enabling detailed analysis of renal microvasculature. This progress is pivotal for investigating renal pathologies and assessing the efficacy of therapeutic interventions, highlighting the potential of SRUS in renal disease studies.

## Outstanding Challenges

The advent of 3D SRUS has significantly advanced the field of medical imaging, offering unprecedented insights into microvascular structures and dynamics at a subwavelength scale. Despite these technological leaps, several challenges persist, impacting the efficacy, applicability, and widespread adoption of this technique. This section outlines the outstanding challenges identified from the reviewed literature, providing a comprehensive understanding of the current limitations and areas requiring further research and development.

### Computational Complexity

While a fully sampled 2D matrix array offers the potential for comprehensive 3D US imaging, clinical implementation faces significant challenges due to the immense computational demands associated with managing the vast amounts of volumetric data involved. The challenge is exacerbated by the usage of complex post-processing algorithms, necessitating powerful computing solutions to manage and process the data within a reasonable timeframe.^20,22^

### Long Acquisition Times

A recurring challenge across studies is the extended acquisition time required for 3D SRUS. For instance, the imaging of microvascular morphology features in tumor angiogenesis necessitates long imaging durations (up to 30 minutes for typical sized rat tumors) due to the need for capturing a large number of frames to ensure sufficient MB detection and microvascular visualization [d]. Acquisition durations of several tens of seconds to minutes, coupled with processing times that can extend to several hours,^29^ limit the practicality of 3D SRUS for real-time clinical applications.

### Signal-to-Noise Ratio

To achieve 3D SRUS imaging with fewer channel counts and reduce the system complexity, researchers have proposed various novel transducer configurations like sparse arrays and row-column arrays. However, they suffer from degraded PSF and a poor signal-to-noise (SNR).^27^ SNR can be enhanced by increasing the number of transmissions, which again results in larger data. Furthermore, the SNR of 2D arrays is fundamentally lower than that of 1D arrays due to the smaller element size, which hinders imaging small microvessels.^21^

### Motion Artifacts During Image Acquisition

Motion artifacts significantly impact the accuracy of 3D SRUS imaging, where tissue movement can distort MB localization, requiring robust motion correction strategies. High-volume imaging rates can help mitigate motion artifacts,^3,15,20,30^ yet managing the large data sizes generated poses substantial challenges. Furthermore, the translation of these techniques to human imaging is complicated by the inability to physically restrain patients as in preclinical models,^27^ necessitating more sophisticated and adaptable motion correction algorithms. Studies indicate substantial data loss, with 20%–30% of frames discarded due to motion-induced artifacts,^17^ emphasizing the need for improved algorithms that can handle complex, dynamic in vivo environments (e.g., while imaging kidney, liver, or heart) effectively.^6^

### MB Dynamics

MB dynamics, such as concentration, velocity, and their interaction with US waves, significantly affect 3D SRUS image quality and necessitate further research.^17^ High MB concentrations enhance localization in smaller microvessels but hinder separability in larger microvascular structures, thereby challenging vascular resolution and requiring novel algorithms for effective discrimination and isolation.^31^ In transcranial applications, it is essential to adapt US pressures to each patient’s skull characteristics to prevent MB destruction.^27^ High insonation rates combined with plane wave US imaging can destroy MB prematurely, impacting both SNR and flow measurement accuracy.^8^ Thus, optimizing PRF, MB flow velocity, imaging pressure, and compounding strategies is critical for improving SRUS across various clinical settings.

### Transducer and Array Design

The design of transducers and arrays for 3D SRUS presents substantial technological challenges. Efforts have been made to investigate row-column addressed matrix arrays, sparse arrays, and multiplexing techniques to reduce the complexity of hardware. Nevertheless, these methods frequently lead to trade-offs in terms of resolution, SNR, and the flexibility of imaging regimens.^8,27^ Future research needs to focus on creating transducer arrays that provide high resolution while also achieving deep penetration, wide field of view, and without adding significant system complexity.

### Clinical Translation and Scalability

Although 3D SRUS approaches have demonstrated encouraging outcomes in preclinical investigations, their use in clinical environments presents numerous obstacles. These factors encompass the requirement for wider field-of-view imaging to meet the structure of the human body, the modification of algorithms and processing methodologies to suit clinical data, the ability of the technology to be easily implemented in regular clinical practice, and non-real-time processing.^33^ It is crucial to ensure that approaches are versatile, easy to use, and compatible with current clinical workflow to facilitate seamless integration with existing healthcare systems.

### Machine Learning Integration

The integration of machine learning (ML) into 3D SRUS presents both challenges and opportunities. ML has been successfully applied in 2D SRUS for tasks including tissue decluttering and MB localization, resulting in a significant reduction in data processing times.^34–37^ Extending ML benefits to 3D SRUS offers a promising solution to the substantial computational demands of this technology. However, applying ML advancements to 3D SRUS is challenging due to the increased complexity and volume of data. Nevertheless, pursuing this exploration is crucial for data-intensive 3D SRUS applications, paving the way for real-time, high-resolution volumetric US imaging in clinical settings.

## Conclusion

The review highlights the versatility and transformative potential of 3D SRUS across a broad spectrum of clinical applications, from enhancing neurovascular and oncological imaging to pioneering noninvasive methods for lymphatic system evaluation and ocular disease diagnosis. The ongoing development and refinement of 3D SRUS techniques promise to further expand use in clinical practice, offering a non-ionizing, cost-effective alternative to traditional medical imaging modalities with the added benefit of superior resolution capability and depth penetration. It is worth noting that many of the reviewed works focus on the technical aspects and validation of 3D SRUS techniques, which are essential for the successful clinical translation and application of these techniques in the aforementioned areas. Nonetheless, several outstanding challenges remain. These include the need for reduced computational complexity, improved SNR, enhanced MB stability, and the integration of 3D SRUS into standardized clinical workflows. Addressing these challenges is essential for maximizing the impact of 3D SRUS in healthcare settings and will likely be a focal point of future research in this domain.

## Figures and Tables

**Figure 1.**
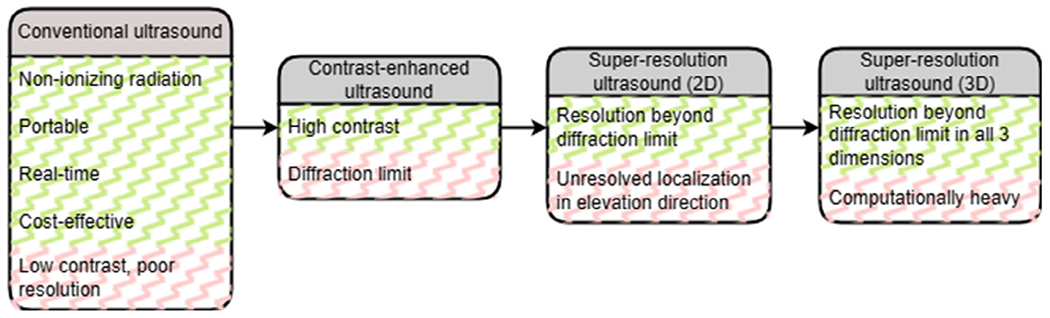
Transition from conventional 2-dimensional (2D) ultrasound (US) techniques to 3-dimensional (3D) super-resolution US (SRUS) imaging.

**Table 1.** Comparison of Key Aspects in 3D Super-Resolution Ultrasound Studies

Reference	Study Subject	Contrast Used	Hardware, Probe Center Frequency, and Imaging Technique	Clutter Filter	Localization Algorithm	Acquisition Time	Spatial Resolution and Flow Velocity
Errico et al^15^	Male Sprague–Dawley rats’ brain, specifically cerebral microvessels	1–5 μm perfluorocarbon-filled MB (Bracco)	Clinical scanner, 256 TX/128 RX channels, 160-element custom array, 20.3 MHz, plane-wave compounding (−3°, 0°, 3°), 500 Hz frame rate	SVD-based high-pass spatiotemporal filter	Deconvolving with Gaussian PSF	10 minutes per each coronal plane of whole brain	10 μm, 15 mm/second at 80 μm diameter and 2 mm/second at 15 μm diameter
Lin et al^17^	Female Fischer 344 rats, subcutaneous fibrosarcoma tumors in the right flank region	Lipid-encapsulated MB containing decafluorobutane, mean diameter 0.9 μm	Vantage with L11-5 probe, plane-wave imaging at 500 Hz frame rate	SVD-based high-pass spatiotemporal filter	Hysteresis thresholding	26 seconds per slice, 11–33 minutes for complete 3D scan	Smallest vessel diameter 25 μm, NA
Zhu et al^18^	New Zealand white rabbits, popliteal lymph nodes	In-house gas-filled MB, mean diameter 1 μm	Vantage (256-channel), L22-14 probe, 15-angle plane waves (−7.5° to 7.5°), 500 Hz frame rate	NA	Comparing features with a PSF and intensity weighted center of mass detection	2.4 seconds for each scan position, total scanning time 40.8 seconds	<80 μm, <5 mm/second
Oezdemir et al^6,19^	Fertilized White Leghorn chicken eggs, developing chicken embryo	Custom MB	Vevo 3100 preclinical scanner, MX250 transducer, 21 MHz, MB-sensitive mode	Singular value filter	8-connected component analysis and centroid detection	152 fps with a total of 2000 frames for each tissue cross-section	Smallest vessel diameter 20–30 μm, NA
Oezdemir et al^16^	Female BALB/C mice, mammary gland (breast cancer cells)	NA	Vevo 3100, MX201 transducer, 15 MHz	Singular value filter	NA	10^4^ frames acquired at 464 fps at each spatial location	NA
Desailly et al^3^	Phantom with microfluidic channels	SonoVue (Bracco)	128-channel Aixplorer scanner, 1.75 MHz matrix array, plane wave imaging, 1000 Hz frame rate	Differential imaging	Fitting the MB signals with an ultrasonic time-of-flight model	NA	Microfluidic channels as small as 40 μm, NA
Oreilly et al^22^	Spiral phantom containing an ex vivo human skullcap	Difinity (Lantheus medical imaging)	Tx array: 128-element hemispherical, 306 kHz, 128-ch system; Rx array: 128-element sparse, 612 kHz, 128-ch system	Subtraction of reference scan from MB measurement	3D Gaussian fitting	1.5 seconds for 445 frames	NA
Foroozan et al^23^	Spiral phantom containing an ex vivo human skullcap	Difinity (Lantheus Medical Imaging)	Tx array: 128-element hemispherical, 306 kHz, 128-ch system; Rx array: 128-element sparse, 612 kHz center frequency, 128-ch system	NA	Two methods: curve fitting MB images with a 3D Gaussian profile, and image deconvolution	NA	NA
Christensen et al^13^	Phantoms mimicking vascular structures	SonoVue (Bracco)	ULA-OP system, LA332 (144-element), plane wave contrast pulse sequences, 400 Hz frame rate	Empirically selected thresholding	Finding peaks in RF data lines to fit hyperbolas laterally, then calculating the signal’s center of mass	15 seconds	1.9 μm in axial and elevational directions, and 11 μm in lateral plane, NA
Harput et al^7^	Microvessel phantom with two tubes in double helix shape	SonoVue (Bracco)	Two synchronized ULA-OP 256 systems, 512-element spiral array, 3.7 MHz, 9-angle plane wave compounding (±10°)	SVD filter	NA	120 seconds for 3000 volumetric ultrasound frames	193 μm at the widest point of a single tube, NA
Harput et al^24^	A wire phantom for motion estimation and a microvessel flow phantom for simulation tests	Simulated MB	Two ULA-OP256 systems, 512-element 2-D sparse array, 3.7 MHz, 9-angle plane wave compounding (±10°)	SVD filter	MB onset detected when axial profile exceeds 3 SD above noise	333 volumes were acquired in 1 second	NA
Harput et al^8^	A wire phantom for motion estimation and a microvessel flow phantom for simulation tests	Simulated MB	Two ULA-OP256 systems, 512-element 2-D sparse array, 3.7 MHz, 9-angle plane wave compounding (±10°)	SVD filter	MB onset detected when axial profile exceeds 3 SD above noise	333 volumes were acquired in 1 second	NA
Harput et al^5^	Microvascular flow phantom with two twisted tubes	SonoVue (Bracco)	Two ULA-OP 256 systems, 512-element spiral array, 3.7 MHz, 500 Hz frame rate	SVD filter	NA	NA	NA, 49.1 mm/second and 50.7 mm/second in two vessels
Christensen et al^4^	Flow phantom	SonoVue (Bracco)	Philips EPIQ7, X5-1 transducer (3040 elements), 1.25 MHz, contrast-enhanced mode	Rolling background subtraction	MB onset detection when the axial profile exceeds 3 SD above noise	11 seconds for each video segment	NA
Jensen et al^9,25^	3D printed micro-phantoms	SonoVue (Bracco)	SARUS scanner, 62 + 62 Row-Column array, 3 MHz, Synthetic Aperture pulse inversion	Averaging the mean value of the first 20 high-resolution volumes (HRVs) and subtracting this from the processed HRVs	Fitting a second order polynomial to the data, then finding its interpolated peak position	154 volumes per second	NA
Jensen et al^26^	3D printed micro-phantoms containing fixated micro-positioned ultrasound scatterers	Phantom scatterers	SARUS scanner, 62 + 62 RCA array, 3 MHz, synthetic aperture imaging, 160 Hz frame rate	Stationary clutter filter	Interpolating peak position by fitting a second order polynomial to the data and then finding its interpolated maximum position	NA	NA
Heiles et al^20^	Tissue-mimicking phantom with bifurcated vessel structures	SonoVue (Bracco)	Four 256-channel systems, 32 × 32 matrix probe, 8 MHz, 4 tilted plane-wave compounding (±7°)	Subtraction of time averaged intensity of each block from volume data (low concentration case), SVD filter (high concentration case)	QuickPALM particle detection in ImageJ	Volume rate of 500 Hz with maximum acquisition time of 12 seconds	Canal edges as small as 230 μm, Velocities as low as 0.5 mm/second in the *z*-direction and 2.5 mm/second in the (*x*, *y*) directions
Lok et al^11^	Flow channel phantom	Diluted MB	Vantage 256 system, 1024 channel 2-D matrix transducer, 8 MHz, 4 subaperture processing, 4-angle compounding, 300 Hz frame rate	3D SVD filter	3D normalized cross-correlation with a system PSF	NA	NA
Lok et al^21^	Flow channel phantom, chicken embryo brain	Lumason MB (mean diameter 1.5–2.5 μm)	Vantage 256 system, UTA 1024 MUX, 1024-channel 2D matrix probe, 9-angle plane-wave imaging	SVD filter	3D normalized cross-correlation with system PSF, followed by regional peak identification	22 seconds for effective imaging after MB injection	Around 52 μm, NA
Yan et al^10^	Phantom with two tubes, and New Zealand male white rabbits’ kidney	In-house perfluorobutane MB	Vantage 256 system, 32 × 32 matrix probe, 4 subaperture multiplexing, 500 Hz frame rate	SVD filter	3D normalized cross-correlation, followed by peaks finding	2 seconds for in vitro and 3 seconds for in vivo	83.3,78.1, 75.2, and 74.1 μm for DAS, p-DAS, CF, and CV beamformers, respectively, NA
Chavignon et al^27,28^	Sprague–Dawley male rat Brain	SonoVue (Bracco)	Vantage 256 with 32 × 32 matrix probe, 7.8 MHz, plane wave transmissions, multiplexed subaperture receptions	SVD filter	3D radial symmetry algorithm	100,000 volumes within 7 minutes	30 μm, Ranging from 4.3 to 28.4 mm/s
Demeulenaere et al^29^	C57Bl/6 male mice brain	SonoVue (Bracco)	4 Vantage 256 systems, 32 × 32 matrix probe at 9 MHz, 16 plane wave transmissions	SVD filter	Correlating with a Gaussian kernel, then fitting with a 3D paraboloid	750 volumes per second, total duration 15 minutes	20 μm, from 2 mm/second in arterioles and venules up to 100 mm/second in larger vessels
		SonoVue (Bracco)		SVD filter	3D paraboloid fitting		
Demeulenaere et al^30^	Sprague–Dawley male rats’ heart		4 Vantage 256 systems, 32 × 32 matrix probe at 9 MHz, plane wave compounding (−5.7° to +5.7°)			2,220 volumes per second, 270 ms for each burst of acquisition	< 20 μm, From 10 mm/second in tiny arterioles up to 300 mm/second in large arteries
McCall et al^31^	Fischer 344 female rats’ brain	In-house formulated MB (mean diameter 1.6 μm)	4 synchronized Vantage 256 systems, 32 × 32 matrix transducer, 7.81 MHz, 5-angle plane-wave compounding	SVD filter	Convolution with system PSF followed by weighted centroid detection	200 seconds at a rate of 500 volumes per second	16 μm, NA
Lei et al^32^	Rabbits’ ocular microvasculature	Sonazoid (GE Healthcare)	Four synchronized Vantage 256 systems, 32 × 32 matrix transducer, 8 MHz, nine-angle plane wave imaging	Block-wise SVD filter	3D cross-correlation with a Gaussian kernel (obtained from simulated PSF)	Acquisition time of 90 seconds for 30 groups of data, each consisting of 300 volumes, collected with an effective imaging rate of 500 volumes/second	54 μm and distinguishing two vessels 73 μm apart, 5 mm/second

NA, not available.

## Data Availability

Data sharing is not relevant to this article as no data sets were generated or analyzed in the present study.

## Abbreviations

1D: 1-dimensional
2D: 2-dimensional
3D: 3-dimensional
CEUS: contrast-enhanced ultrasound
CT: computed tomography
MB: microbubble
MRI: magnetic resonance imaging
PSF: point spread function
ROI: region of interest
SRUS: super-resolution ultrasound
SVD: singular value decomposition
ULM: US localization microscopy
US: ultrasound
VDA: vascular disrupting agent

## References

[1] Chen Q, Song H, Yu J, Kim K. Current development and applications of super-resolution ultrasound imaging. Sensors 2021; 21:2417. 33915779 10.3390/s21072417 PMC8038018

[2] Kaffas AE, Vo-Phamhi JM, Griffin JF, Hoyt K. Critical advances for democratizing ultrasound diagnostics in human and veterinary medicine. Annu Rev Biomed Eng 2024; 26:49–65. 38166185 10.1146/annurev-bioeng-110222-095229 PMC11238906

[3] Desailly Y, Couture O, Fink M, Tanter M. Sono-activated ultrasound localization microscopy. Appl Phys Lett 2013; 103:174107.

[4] Christensen-Jeffries K, Harput S, Brown J, 3D in vitro ultrasound super-resolution imaging using a clinical system. Paper presented at: 2018 IEEE International Ultrasonics Symposium (IUS); 22-25 October 2018; Kobe, Japan.

[5] Harput S, Toulemonde M, Ramalli A, Quantitative microvessel analysis with 3D super-resolution ultrasound and velocity mapping. Paper presented at: 2020 IEEE International Ultrasonics Symposium (IUS); 07-11 September 2020; Las Vegas, NV, USA.

[6] Özdemir I, Johnson K, Mohr-Allen S, Peak KE, Varner V, Hoyt K. Three-dimensional visualization and improved quantification with super-resolution ultrasound imaging—validation framework for analysis of microvascular morphology using a chicken embryo model. Phys Med Biol 2021; 66:085008. 10.1088/1361-6560/abf203 PMC8463964 33765676

[7] Harput S, Christensen-Jeffries K, Ramalli A, 3-D super-resolution ultrasound imaging using a 2-D sparse array with high volumetric imaging rate. Paper presented at: 2018 IEEE International Ultrasonics Symposium (IUS); 22-25 October 2018; Kobe, Japan.

[8] Harput S, Christensen-Jeffries K, Ramalli A, 3-D super-resolution ultrasound imaging with a 2-D sparse array. IEEE Trans Ultrason Ferroelectr Freq Control 2020; 67:269–277. 10.1109/TUFFC.2019.2943646. 31562080 PMC7614008

[9] Jensen JA, Ommen ML, Oygard SH, Three-dimensional super-resolution imaging using a row–column array. IEEE Trans Ultrason Ferroelectr Freq Control 2020; 67:538–546. 10.1109/TUFFC.2019.2948563. 31634831

[10] Yan J, Wang B, Riemer K, Fast 3D super-resolution ultrasound with adaptive weight-based beamforming. IEEE Trans Biomed Eng 2023; 70:2752–2761. 37015124 10.1109/TBME.2023.3263369 PMC7614997

[11] Lok U-W, Huang C, Tang S, Three-dimensional super-resolution ultrasound microvessel imaging with bipartite graph-based microbubble tracking using a verasonics 256-channel ultrasound system. Paper presented at: 2019 IEEE International Ultrasonics Symposium (IUS); 06-09 October 2019; Glasgow, UK.

[12] Huang Q, Zeng Z. A review on real-time 3D ultrasound imaging technology. Biomed Res Int 2017; 2017:1–20. 10.1155/2017/6027029 PMC5385255 28459067

[13] Christensen-Jeffries K, Brown J, Aljabar P, Tang M, Dunsby C, Eckersley RJ. 3-D in vitro acoustic super-resolution and super-resolved velocity mapping using microbubbles. IEEE Trans Ultrason Ferroelectr Freq Control 2017; 64:1478–1486. 28767367 10.1109/TUFFC.2017.2731664

[14] Mohamed F, Siang CV. A survey on 3D ultrasound reconstruction techniques. In: Aceves-Fernandez Marco Antonio (ed). Artificial Intelligence - Applications in Medicine and Biology. London, UK: IntechOpen; 2019.

[15] Errico C, Pierre J, Pezet S, Ultrafast ultrasound localization microscopy for deep super-resolution vascular imaging. Nature 2015; 527:499–502. 10.1038/nature16066. 26607546

[16] Oezdemir I, Li J, Song J, Hoyt K. 3-D super-resolution ultrasound imaging for monitoring early changes in breast cancer after treatment with a vascular-disrupting agent. Paper presented at: 2021 IEEE International Ultrasonics Symposium (IUS); 11-16 September 2021; Xi’an, China. 10.1109/IUS52206.2021.9593426 PMC10863700 38351971

[17] Lin F, Shelton SE, Espíndola D, Rojas JD, Pinton G, Dayton PA. 3-D ultrasound localization microscopy for identifying microvascular morphology features of tumor angiogenesis at a resolution beyond the diffraction limit of conventional ultrasound. Theranostics 2017; 7:196–204. 28042327 10.7150/thno.16899 PMC5196896

[18] Zhu J, Rowland EM, Harput S, 3D super-resolution US imaging of rabbit lymph node vasculature in vivo by using microbubbles. Radiology 2019; 291:642–650. 10.1148/radiol.2019182593. 30990382

[19] Oezdemir I, Mohr-Allen S, Peak KE, Varner V, Hoyt K. Three-dimensional super-resolution ultrasound imaging of chicken embryos—a validation framework for analysis of microvascular morphology. Paper presented at: 2020 IEEE International Ultrasonics Symposium (IUS); 07-11 September 2020; Las Vegas, NV, USA. 10.1109/ius46767.2020.9251486 PMC9744579 36514782

[20] Heiles B, Correia M, Hingot V, Ultrafast 3D ultrasound localization microscopy using a 32 × 32 matrix array. IEEE Trans Med Imaging 2019; 38:2005–2015. 30946662 10.1109/TMI.2018.2890358

[21] Lok U-W, Huang C, Trzasko JD, Three-dimensional ultrasound localization microscopy with bipartite graph-based microbubble pairing and Kalman-filtering-based tracking on a 256-channel verasonics ultrasound system with a 32 × 32 matrix array. J Med Biol Eng 2022; 42:767–779. 10.1007/s40846-022-00755-y. 36712192 PMC9881453

[22] O’Reilly MA, Hynynen K. A super-resolution ultrasound method for brain vascular mapping. Med Phys 2013; 40:110701. 24320408 10.1118/1.4823762 PMC3799687

[23] Foroozan F, O’Reilly MA, Hynynen K. Microbubble localization for three-dimensional Super-resolution ultrasound imaging using curve fitting and deconvolution methods. IEEE Trans Biomed Eng 2018; 65:2692–2703. 29993387 10.1109/TBME.2018.2813759 PMC6459186

[24] Harput S, Christensen-Jeffries K, Brown J, 3-D motion correction for volumetric super-resolution ultrasound imaging. Paper presented at: 2018 IEEE International Ultrasonics Symposium (IUS); 22-25 October 2018; Kobe, Japan. 10.1109/ULTSYM.2018.8580145 PMC7610905 34093969

[25] Jensen JA, Tomov B, Schou M, 3-D super resolution imaging using a 62 + 62 elements row-column Array. Paper presented at: 2019 IEEE International Ultrasonics Symposium (IUS); 06-09 October 2019; Glasgow, UK.

[26] Ommen ML, Schou M, Beers C, Jensen JA, Larsen NB, Thomsen EV. 3D printed calibration micro-phantoms for super-resolution ultrasound imaging validation. Ultrasonics 2021; 114:106353. 33721683 10.1016/j.ultras.2021.106353

[27] Chavignon A, Heiles B, Hingot V, Orset C, Vivien D, Couture O. 3D transcranial ultrasound localization microscopy in the rat brain with a multiplexed matrix probe. IEEE Trans Biomed Eng 2022; 69:2132–2142. 34932470 10.1109/TBME.2021.3137265

[28] Chavignon A, Hingot V, Orset C, Vivien D, Couture O. 3D transcranial ultrasound localization microscopy for discrimination between ischemic and hemorrhagic stroke in early phase. Sci Rep 2022; 12:14607. 36028542 10.1038/s41598-022-18025-x PMC9418177

[29] Demeulenaere O, Bertolo A, Pezet S, In vivo whole brain microvascular imaging in mice using transcranial 3D ultrasound localization microscopy. EBioMedicine 2022; 79:103995. 10.1016/j.ebiom.2022.103995. 35460988 PMC9048085

[30] Demeulenaere O, Sandoval Z, Mateo P, Coronary flow assessment using 3-dimensional ultrafast ultrasound localization microscopy. JACC Cardiovasc Imaging 2022; 15:1193–1208. 10.1016/j.jcmg.2022.02.008. 35798395

[31] McCall JR, Santibáñez F, Belgharbi H, Pinton G, Dayton PA. Non-invasive transcranial volumetric ultrasound localization microscopy of the rat brain with continuous, high volume-rate acquisition. Theranostics 2023; 13:1235–1246. 36923540 10.7150/thno.79189 PMC10008741

[32] Lei S, Zhang C, Zhu B, In vivo ocular microvasculature imaging in rabbits with 3D ultrasound localization microscopy. Ultrasonics 2023; 133:107022. 37178486 10.1016/j.ultras.2023.107022

[33] Song P, Rubin JM, Lowerison MR. Super-resolution ultrasound microvascular imaging: is it ready for clinical use? Z Für Med Phys 2023; 33:309–323. 10.1016/j.zemedi.2023.04.001 PMC10517403 37211457

[34] Brown K, Ghosh D, Hoyt K. Deep learning of spatiotemporal filtering for fast super-resolution ultrasound imaging. IEEE Trans Ultrason Ferroelectr Freq Control 2020; 67:1820–1829. 32305911 10.1109/TUFFC.2020.2988164 PMC7523282

[35] Brown K, Waggener SC, Redfern AD, Hoyt K. Faster super-resolution ultrasound imaging with a deep learning model for tissue decluttering and contrast agent localization. Biomed Phys Eng Express 2021; 7:065035. 10.1088/2057-1976/ac2f71 PMC8594285 34644679

[36] Brown K, Dormer J, Fei B, Hoyt K. Deep 3D convolutional neural networks for fast super-resolution ultrasound imaging. SPIE Med Imaging 2019; 10955:1095502. 10.1117/12.2511897 PMC7261615 32476699

[37] Brown K, Waggener SC, Redfern AD, Hoyt K. Deep learning implementation of super-resolution ultrasound imaging for tissue decluttering and contrast agent localization. Paper presented at: 2020 IEEE International Ultrasonics Symposium (IUS); 07-11 September 2020; Las Vegas, NV, USA.

